# Anti-tumor Activity of Toll-Like Receptor 7 Agonists

**DOI:** 10.3389/fphar.2017.00304

**Published:** 2017-05-31

**Authors:** Huju Chi, Chunman Li, Flora Sha Zhao, Li Zhang, Tzi Bun Ng, Guangyi Jin, Ou Sha

**Affiliations:** ^1^Department of Anatomy, Histology and Developmental Biology, School of Basic Medical Sciences, Shenzhen University Health Science CentreShenzhen, China; ^2^School of Life Sciences, Faculty of Science, The Chinese University of Hong KongHong Kong, Hong Kong; ^3^Department of Physiology and Neurology, University of ConnecticutStorrs, CT, United States; ^4^Departmet of Biochemistry, Faculty of Science, The Chinese University of Hong KongHong Kong, Hong Kong; ^5^Department of Pharmacy, Shenzhen University Health Science CentreShenzhen, China

**Keywords:** Toll-Like receptors (TLRs), TLR7, agonists, anti-tumor activity, immune stimulation

## Abstract

Toll-like receptors (TLRs) are a class of pattern recognition receptors that play a bridging role in innate immunity and adaptive immunity. The activated TLRs not only induce inflammatory responses, but also elicit the development of antigen specific immunity. TLR7, a member of TLR family, is an intracellular receptor expressed on the membrane of endosomes. TLR7 can be triggered not only by ssRNA during viral infections, but also by immune modifiers that share a similar structure to nucleosides. Its powerful immune stimulatory action can be potentially used in the anti-tumor therapy. This article reviewed the anti-tumor activity and mechanism of TLR7 agonists that are frequently applied in preclinical and clinical investigations, and mainly focused on small synthetic molecules, including imiquimod, resiquimod, gardiquimod, and 852A, etc.

## Introduction

Toll-like receptors (TLRs) are a class of pattern recognition receptors that play a bridging role in innate immunity and adaptive immunity (O'Neill et al., 2013). TLRs can recognize both pathogen-associated molecular patterns and damage-associated molecular patterns such as lipopolysaccharide and free nucleic acids (Piccinini and Midwood, 2010). Normally, TLRs are expressed by macrophages, dendritic cells, natural killer (NK) cells and epithelial cells. Some TLRs are expressed in the intracellular endosomes (TLR3, 7, 8, and 9), while others are localized on the plasmalemma (TLR1, 2, 4, 5, 6, 10, and 11; Hennessy et al., 2010). TLRs are activated by diverse agonists, e.g., TLR4 by lipopolysaccharide, and TLR3, 7 and 9 by nucleic acids (Table 1). The binding of a TLR and its agonist generates an immune response, important for therapeutic research, including research on anti-cancer therapy. TLR agonist-based cancer immunotherapy has been used in preclinical and clinical investigations (Vacchelli et al., 2013). Most of the TLR agonists are clinically tolerated and biologically active, but some common adverse events were observed. The development of many TLR agonists has been discontinued in consequence of lacking efficacy in phase III trial (Galluzzi et al., 2012). Among TLRs, TLR7 is an intracellular receptor expressed on endosomal membranes. TLR7 is closely related to TLR8, which also recognizes nucleosides and nucleotides from intracellular pathogens. There are two ligand-binding sites in TLR7. The first site for binding of small ligands is conserved in both TLR7 and TLR8. The second site differs from that of TLR8, and is used to bind with ssRNA to enhance activation of the first site (Maeda and Akira, 2016). Activation of TLR7 can induce Type 1 interferon and inflammatory response, therefore targeting TLR7 is a promising strategy for both antiviral and anti-tumor therapy. This article aims to review the anti-tumor activity of TLR7 agonists with a focus on small synthetic molecules.

**Table 1:** **Summary of the anti-tumor activity of TLRs**.

**TLR**	**Agonist**	**Tumor type**	***In vitro***	***In vivo***	**References**
1/2	Pam3Cys-SK4	Melanoma	B16F10	C57BL/6	Stone et al., 2009b; Oldford et al., 2010
		Gliomas	GL261	C57BL/6	Grauer et al., 2008
	BLP	Lung cancer	3LL	C57BL/6	Zhang et al., 2011
		Leukemia	FBL3	C57BL/6	Zhang et al., 2011
		Melanoma	F10	C57BL/6	Zhang et al., 2011
2/6	Pam2CSK4	Leukaemia	WEHI-3B	BALB/c	Shcheblyakov et al., 2011
3	poly(I:C)	Melanoma	B16 SK-MEL-13, -28, -37	C57BL/6 NOD/SCID mice	Chiba et al., 2013; Le Noci et al., 2015
		Gastric carcinoma	AGS, BGC-823,	BALB/c nude mice	Qu et al., 2013
		Bladder cancer	MBT-2	C3H mice	Ayari et al., 2016
		Mesothelioma	AB1	BALB/c	Stone et al., 2009a
		Hepatic carcinoma	HepG2.2.15	Rat	Chen et al., 2012; Xu et al., 2013
		Prostate Cancer	DU145TRAMP-C1, MDA-MB-231, PC3	C57BL/6	Paone et al., 2010; Galli et al., 2013
4	MPL	Cervical cancer	TC-1	C57BL/6	Gableh et al., 2016
	E6020	Melanoma	B16BL6 D5-HER2	C57BL/6 SCID	Davis et al., 2011; Wang et al., 2012
	LPS	Colon cancer	CT-26	BALB/c	Pham et al., 2010; Davis et al., 2011
		Squamous carcinoma	SCCFVII/SF	BALB/c	Davis et al., 2011
	RG-II	Lymphoma	EL-4	C57BL/6	Park et al., 2013
5	CBLB502	Lymphoma	RMAS	C57BL/6	Leigh et al., 2014
		Lung Cancer	A549	athymic nu/nu mice	Zhou et al., 2012
	MAP1S	Breast cancer	MCF-7, MDA-MB-435s, MDA-MB-468, T47D, MDA-MB-231	/	Zhou et al., 2012
7	Imiquimod	Squamous carcinoma	YD-10B, FaDu,	/	Ahn et al., 2012
		Prostate cancer	TRAMP-C2, PC3	C57BL/6	Han et al., 2013
		Bladder cancer	MB49	C57BL/6	Hayashi et al., 2010
		Breast cancer	TSA	BALB/c Human (preclinical)	Adams et al., 2012; Dewan et al., 2012
		Melanoma	/	Human (preclinical)	Narayan et al., 2012
	Resiquimod	Gliomas	GL261	C57BL/6	Grauer et al., 2008
		Acute myeloid leukemia	HL60, THP1, OCI-AML3, HCT116, 293T	Nod/SCID/IL2Rγ-/-(NSG)	Smits et al., 2010; Ignatz-Hoover et al., 2015
		Breast cancer	4T1	BALB/c	Yin et al., 2015
		T-cell lymphoma	/	Human (phase I)	Rook et al., 2015
	Gardiquimod	Melanoma	B16	C57BL/6	Ma et al., 2010
		Pancreatic cancer	BxPC-3	/	Zou et al., 2015
7	852A	Ovarian cancers	/	Human (preclinical)	Geller et al., 2010
		Cervix cancer	/	Human (preclinical)	Geller et al., 2010
		Breast cancer	/	Human (preclinical)	Geller et al., 2010
		Melanoma	/	Human (phase II)	Dummer et al., 2008
		lymphocytic leukemia	/	Human (phase I/II)	Spaner et al., 2010
	Loxoribine	Melanoma	B16	C57BL/6	Pope et al., 1994
		B-chronic leukemia	/	Human (preclinical)	Tosi et al., 1997; Pellacani et al., 1999
	Bropirimine	Bladder tumor	KK-47 724	/	Tei et al., 2002
		Prostate cancer	MBT-2	/	Sarosdy, 1997
		Renal-cell carcinoma	Renca	BALB/c	Fujioka et al., 1995
	3M-011	Pancreatic cancer	BxPC3 Panc-1	BALB/c C57Bl/6	Scholch et al., 2015
		Colon cancer	HT29 HCT-116	BALB/c C57Bl/6	Scholch et al., 2015
	3M-052	Melanoma	B16.F10, B16.OVA, BP	C57BL/6	Singh et al., 2014
	DSR-6434	Colon cancer	CT26	C3H BALB/c	Adlard et al., 2014
		Renal cell carcinoma	Renca	Balb/c C57BL/6	Koga-Yamakawa et al., 2015
	DSR-29133	Colon cancer	CT26	Balb/c	Dovedi et al., 2016
		Osteosarcoma	LM8	C3H	Dovedi et al., 2016
		Renal cell carcinoma	Renca	Balb/c	Dovedi et al., 2016
	SC1	Lymphoma	RMA-S	C57BL/6	Wiedemann et al., 2016
		Renal cell carcinoma	Renca	Balb/c	Hamm et al., 2009
	SZU-101	Breast carcinoma	4T1	Balb/c	Diao et al., 2016
		Gastric cancer	EAC	Balb/c	Wang et al., 2015
		T cell lymphoma	EL4	C57BL/6	Zhu et al., 2015
	SM-360320	Colon cancer	MC38	BALB/c	Dharmapuri et al., 2009
	SM-276001	Renal cell carcinoma	Renca	Balb/c	Koga-Yamakawa et al., 2013
		Colon cancer	CT26	Balb/c	Koga-Yamakawa et al., 2013
8	Resiquimod	Gliomas	GL261	C57BL/6	Grauer et al., 2008
		Acute myeloid leukemia	HL60, THP1, OCI-AML3, HCT116, 293T	Nod/SCID/IL2Rγ-/-(NSG)	Smits et al., 2010; Ignatz-Hoover et al., 2015
		Breast cancer	4T1	BALB/c	Yin et al., 2015
		T-cell lymphoma	/	Human (phase I)	Rook et al., 2015
	VTX-2337	Lymphoma	/	Human (phase I)	Northfelt et al., 2014
	3M-011	Pancreatic cancer	BxPC3 Panc-1	BALB/c C57Bl/6	Scholch et al., 2015
		Colon cancer	HT29 HCT-116	BALB/c C57Bl/6	Scholch et al., 2015
9	CpG-ODN	Gliomas	GL261	C57Bl/6	Grauer et al., 2008
		Melanoma	B16	C57BL/6	Le Noci et al., 2015
		Lung cancer	95C, 95D	BALB/c	Ren et al., 2009
		Mesothelioma	AB1	BALB/cByJ	Stone et al., 2009a
		B cell lymphoma	/	C57BL/6	Pradhan et al., 2014
		T-cell lymphoma	/	Human (phase I)	Kim et al., 2010
		Lymphoma	/	Human (Phase I/II)	Brody et al., 2010
		Neoplastic meningitis	/	Human (phase I)	Ursu et al., 2015
		Hepatic carcinoma	HepG2, H7402, PLC/PRF/5	athymic nu/nu mice	Zhang et al., 2014
	IMO	Colon cancer	GEO SW48 LS174T	BALB/cAnNCrlBR athymic (nu/nu) mice	Damiano et al., 2006, 2007; Conforti et al., 2010; Rosa et al., 2011
		Pancreatic Cancers	AsPC1/GLT	BALB/cAnNCrlBR athymic (nu/nu) mice	Rosa et al., 2011
		Non-small cell lung cancer	/	Human (phase II)	Smith et al., 2014
		Breast Carcinoma	/	BALB/c	Aurisicchio et al., 2009
	1018 ISS	Lymphoma	/	Human (phase II)	Friedberg et al., 2009

LPS, Lipopolysaccharide; BLP, Bacterial lipoprotein; Poly (I:C), Polyinosinic-polycytidylic acid; MPL, Monophosphoryl lipid A; RG II, Rhamnogalacturonan II;

*IMO, Immune modulatory oligonucleotide*.

## Imiquimod

Imiquimod, also called Aldara or R-837, is an immune response modifier acting as a TLR7 agonist. It has been approved by Food and Drug Administration (FDA) as a therapeutic agent for basal cell carcinoma and genital warts (Vacchelli et al., 2012). The structure of imiquimod is similar to adenosine nucleoside and it can interact with adenosine receptors. Imiquimod has now been well studied and more and more research is focusing on its anti-tumor activities. TLR7 is highly expressed in oral squamous cell carcinoma cells. The growth of these cells can be significantly inhibited and apoptosis through mitochondria-dependent pathway brought about by treatment with imiquimod. (Ahn et al., 2012). From another aspect, effector T cells from imiquimod-treated squamous cell carcinoma cells generate more IFN-γ and less IL-10 compared with untreated cells (Huang et al., 2009). Imiquimod inhibits the proliferation and also arrests the cell cycle in both murine and human prostate cancer cells (Han et al., 2013).

However, the anti-tumor activities of imiquimod can be counteracted by immunosuppressive cytokines and other molecules, such as IL-10, indoleamine 2,3-dioxygenase (IDO) and induced nitric oxide synthase. IDO expression is upregulated by imiquimod. Therefore, different kinds of combinational therapy with imiquimod have been used to overcome the shortcoming of using imiquimod alone. Ito et al. (2015a) found that combining imiquimod with 1-methyl-D-tryptophan, an IDO inhibitor, largely inhibited the growth of tumor cells, and enhanced the efficacy of imiquimod through induction of Th1 response. Blocking of IL-10 and nitric oxide synthase was also applied to combination therapy using imiquimod (Lu et al., 2010; Ito et al., 2015b). In a combined chemotherapy in phase II clinical trial, breast cancer patients were treated with imiquimod plus albumin-bound paclitaxel, disease regression was induced and 92% of adverse events were grade 1 and grade 2. However, the responses were short-lived (Salazar et al., 2017). Using TLR agonists as chemoadjuvants decreased the possibility of adverse events and enhanced the efficiency of chemical agents with a diminished dosage, but survival prospects were still modest (Ding et al., 2017).

Imiquimod is slightly soluble in common solvents and insoluble in water. Hence poloxamer polymer was added to prolong local contact and lessen systemic absorption of imiquimod. Incorporating 2-(hydroxypropyl)-β-cyclodextrin in the formulation should augment the physical stability, and a clear homogeneous solution was produced. This kind of combination enhanced chemokine induction and showed anti-tumor effects in an orthotopic mouse model of bladder cancer (Hayashi et al., 2010). Local imiquimod treatment induced a systemic antigen-specific CD8 response, but did not prevent the growth of distal tumor because of a lack of CD4 T cell response. Combining local imiquimod with anti-CD40 therapy reinforced the local response, and upregulated the ratio of regression of distal tumor (Broomfield et al., 2009; Dewan et al., 2012) found that imiquimod inhibited the growth of cutaneous breast cancer cells by a CD8 dependent mechanism, but did not cause complete tumor regression. In addition, the effects of imiquimod were abolished with depletion of CD8+ T cells. Synergistic effects of local radiotherapy and imiquimod could rectify this situation, and a low dose of cyclophosphamide further enhanced these effects, and reduced recurrence by inducing protective immunological memory.

Imiquimod is an FDA-approved TLR agonist that has aroused considerable clinical research interest, and showed promising results in clinical studies. In seven imiquimod treated BCC patients, complete histopathological tumor clearance was observed 6 weeks after initiation of treatments and no signs of recurrence were detected. Most importantly, systemic side effects such as myalgia, lymphadenopathy were indiscernible during the trial (Love et al., 2016). In patients with genital warts, the serum concentrations were low after daily treatment of imiquimod. There were no serious adverse events, indicating the safety of imiquimod (Wu et al., 2012). Moreover, imiquimod can promote a pro-immunogenic tumor environment. Melanoma metastases often fail to respond to immune therapies because of the lack of T cells. Based on immune activation, imiquimod could be an efficacious therapeutic agent against melanoma (Narayan et al., 2012). Four patients, treated with imiquimod daily and immunized with a vaccine consisting of a tetanus toxoid-derived helper peptide and 12 melanom peptides, showed increased expression of cytokines and chemokines as well as CD8+T cell infiltrates (Mauldin et al., 2016).

## Resiquimod and gardiquimod

Both resiquimod and gardiquimod have an imidazoquinoline structure, share a similar structure with imiquimod, but have more potential properties than imiquimod.

Targeting tumor angiogenesis has become a prospective strategy for treating cancer in view of the important role of angiogenesis in tumor proliferation. However, a lot of studies have pointed out that the use of anti-angiogenic agent may lead to immunosuppression. So combination therapy is necessitated. Resiquimod exhibited a robust anti-tumor activity in a mouse breast cancer model, combining resiquimod with sunitinib, an antineoplastic agent, largely inhibited the growth of breast cancer cells, and attenuated the immunosuppressive effects of sunitinib (Yin et al., 2015). Cutaneous T-cell lymphoma is malignant tumor of the immune system caused by a mutation of T cells. The malignant T cells migrate to the skin and cause lesions. There is no cure in addition to preventing transplantation. Twelve patients were treated with topical resiquimod gel in a phase I trial. The data revealed that 75% of the patients had improved lesions and 30% of the patients had all lesions cleared. T-cell receptor sequencing demonstrated a decrease of malignant T cells in 90% of the patients and complete elimination in 30% of patients (Rook et al., 2015). It was reported that resiquimod could induce apoptosis of acute myeloid leukemia cells and upregulate the expression of MHC molecules on membranes of acute myeloid leukemia cells. Furthermore, the production of cytokines IL-6, IFN-γ and TNF-α was distinctly elevated (Smits et al., 2010). Resiquimod in combination with radiation therapy, induced expansion of antigen-specific CD8+T cells and prolonged the survival of T cell lymphoma tumor-bearing mice (Dovedi et al., 2013). Resiquimod also has numerous applications as immune adjuvants. Upon treatment with NY-ESO-1, a widely used tumor antigen for vaccination, and imiquimod, CD4+T cell responses but not CD8+T cell responses could be observed in melanoma patients. Thus, Sabado et al. combined another TLR7 agonist, resiquimod, with NY-ESO 1 in treating patients with resected high risk melanoma, and found that CD8+T cell response was increased in a small subset of patients (Sabado et al., 2015). Human papillomavirus (HPV) type 16 is associated with the generation of cervical cancer. Nevertheless, Langerhans cells which serve as antigen presenting cells in the viral infection failed to induce T cell immune response when exposed to HPV16. Resiquimod activated Langerhans cells exposed to HPV16 and induced a specific CD8+ T cell response (Fahey et al., 2009). Oral administration of 0.01 mg/kg resiquimod was tolerated, but serious adverse events were observed at 0.02 mg/kg in patients with hepatitis C virus infection. The 0.02 mg/kg dose of resiquimod leads to IFN-like side effects. Therefore, further studies are necessary to demonstrate the efficacy and safety of TLR7 agonists (Pockros et al., 2007).

Gardiquimod has also been used in cancer therapy. It exhibited a series of potential benefits in oncotherapy, inhibition of cell proliferation, triggering of apoptosis, and suppression of metastasis etc. (Ma et al., 2010; Weber et al., 2013; Zou et al., 2015). These results suggest that the imidazoquinoline family is promising for application in clinical cancer therapy.

## 852A

852A, a TLR7-specific agonist, is more potent and selective than imiquimod. Harrison et al. (2007) evaluated the bioavailability, pharmacokinetics and pharmacodynamics of 852A in a phase I trial by employing different ways of drug delivery. Eighteen healthy adult volunteers were enrolled in the trial. Pharmacokinetic parameters revealed that the subcutaneous route with a bioavailability of 80.5 ± 12.8% was a promising route of administration for subsequent evaluation. Serum concentrations of TNF-α and C-reactive protein were upregulated. Since 852A has a potent immunostimulating function, Geller et al. (2010) explored the anti-tumor activities of 852A in patients with recurrent ovarian cervix and breast cancers. Fifteen patients received 852A subcutaneously for 12 weeks. Sustained tolerability was observed and clinical benefit was modest. The same prolonged subcutaneous administration has also been applied to the treatment of recurrent hematologic malignancies, and demonstrated measurable immune activation (Weigel et al., 2012).

The anti-melanoma activity of 852A was explored in a phase II trial. Patients with chemotherapy-refractory metastatic melanoma received an intravenous injection of 852A which caused systemic immune activation and disease stabilization, even though objective clinical responses were not observed (Dummer et al., 2008). 852A is regarded as a perfect tool for studying the molecular processes of TLR7 in plasmacytoid dendritic cells (pDC). It stimulates pDC for the production of IFNα, and the inhibition of cell proliferaton also depends on pDC and type 1 IFN (Inglefield et al., 2008).

## Other agonists

Numerous studies have focused on synthesizing a variety of small molecules to serve as agonists of TLR7. Loxoribine (7-allyl-8-oxoguanosine) enhanced NK cells activity and induced production of cytokines such as IFNs. It is expected to be helpful to cancer therapy clinically (Agarwala et al., 2000). Bropirimine (2-amin-5-bromo-6-phenyl-4(3)-pyrimidinone) is an orally administered modulator used against renal cell carcinoma in the clinical setting and could induce production of cytokines including IFN-α (Sarosdy et al., 2005). GS-9620 [8-(3-(pyrrolidin-1-ylmethyl) benzyl)-4-amino-2-butoxy-7,8-dihydropteridin-6(5H)-one] is an eligible agonist of TLR7 for the treatment of chronic hepatitis B viral infection. Additionally, oral administration of GS-9620 manifested an antiviral activity without the adverse effects characteristic of systemic response to IFN-α. (Lanford et al., 2013; Fosdick et al., 2014; Bam et al., 2017). 3M-052 is an insoluble injectable TLR7/8 agonist. Intratumoral injection of 3M-052 induced systemic anti-tumor activity and inhibited both local and distal tumor growth in mice bearing wild type B16.F10 melanoma. 3M-052 combined with a checkpoint inhibitor could considerably enhance the effects (Singh et al., 2014). Similarly, 3M-011 boosted the antigen-presenting activities of DC as an adjuvant to radiation therapy. This kind of combination therapy induced local and systemic responses in pancreatic cancer mouse models (Scholch et al., 2015). DSR-6434 has higher water solubility and is more potent toward TLR7 than 852A as mentioned above. Systemic administration of DSR-6434 may reinforce the effect of radiation therapy of cancer in mouse models. However, upon administration twice a week, TLR tolerance emerged and no anti-tumor activity was observed compared with administration once a week, suggesting that activation of DSR-6434 occurs in a dose-dependent manner (Nakamura et al., 2013; Adlard et al., 2014; Koga-Yamakawa et al., 2015). Analogous molecules such as DSR-29133 also have similar potential and the anti-tumor effects can be fortified by combining with low-dose fractionated radiation therapy (Dovedi et al., 2016). Furthermore, SC1, a small molecule agonist of TLR7, has been demonstrated to stimulate NK cells and therefore mediate efficient immune responses, and showed an effective anti-metastatic activity *in vivo*. More specifically, mice bearing NK cell sensitive RMA-S lymphoma were cured by repetitive subcutaneous injections of SC1, and no toxicity or recurrence was observed (Hamm et al., 2009; Wiedemann et al., 2016).

In addition, combining a TLR7 agonist with doxorubicin could be a promising treatment for T cell lymphoma (Zhu et al., 2015). The novel TLR7 agonist SZU-101 which was synthesized in ShenZhen University was also applied to immune adjuvant and acquired prospective outcomes (Wang et al., 2015; Diao et al., 2016). There are also many TLR7 agonists attracting researchers' interest: SM-276001 and SM -360320 are selective TLR7 agonist, and SM-360320 can synergize with DNA vaccines targeting CEA colon cancer and HER2 breast cancer (Dharmapuri et al., 2009; Koga-Yamakawa et al., 2013).

## Anti-tumor mechanism

**Figure 1:**
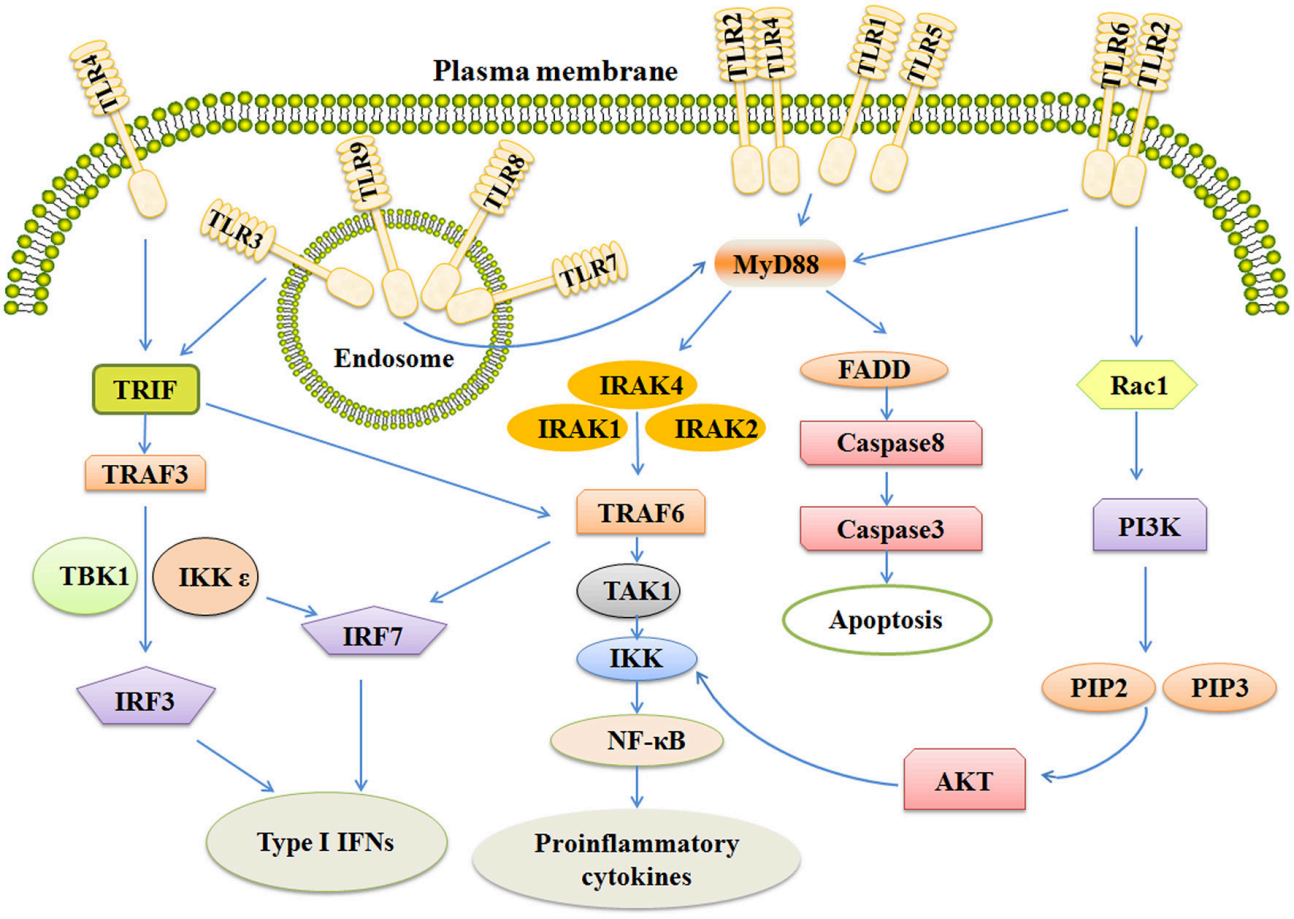
**The signaling pathways of TLRs include MYD-88 independent and MYD-88 dependent pathways**. Activation of TLR3 and TLR4 rely on MYD88 independent pathway which can activate IRF3 and IRF7, together leading to the induction of Type 1 IFNs. MYD-88 dependent pathway ultimately activates NF-κB and IRF7, inducing secretion of IFNs and some pro-inflammatory cytokines. Stimulation of TLR also activates the apoptosis pathway and PI3K/AKT pathway. TRIF, Toll-like receptor adapter molecule; TRAF6, TNF receptor-associated factor 6; IRAK, Interleukin-1 receptor-associated kinase; NF-κB, Nuclear factor κB; IFN, Interferon.

After binding ligands, TLRs change structure for the recruitment of myeloid differentiation primary-response protein 88(MYD88) and TIR-domain-containing adaptor- inducing interferon-β (TRIF). The pathways are MYD88-dependent and MYD88-independent. MyD88 has an amino (N)-terminal death domain (DD), a carboxy (C)-terminal TIR domain, and an intermediate domain which is crucial for TLR signaling (Zou et al., 2016). In the MYD88-dependent pathway, MYD88 complexes with IL-1R-associated kinases 4 (IRAK4) in turn interact with IRAK1 and IRAK2. This kind of association is based on a DD-DD interaction. IRAK4 leads to phosphorylation of IRAK1 and IRAK2, thus promoting its association with tumor necrosis factor (TNF) R-associated factor 6 (TRAF6). TRAF6 is an E3 ubiquitin ligase which takes part in the phosphorylation of transforming growth factor beta-activated kinase 1 (TAK1). Subsequently, these complexes on the one hand activate nuclear factor kappa B (NF-κB), on the other hand, leading to the translocation of Interferon regulatory factor 7 (IRF7; Figure 1).

All TLRs, except TLR3, utilize MYD88-dependent pathway. (Carvalho et al., 2016; Higgins et al., 2016; Martino et al., 2016). TLR3 needs another adapter, TIR-domain-containing adaptor- inducing interferon-β (TRIF). TRIF activates two pathways via TRAF3 and TRAF6 (Wei et al., 2017). TRAF6 next activates TAK1 which can activate IKK complexes, thus leading to the MYD88-independent activation of NF-κB. TRAF3 activates TBK1 and IKKε, ultimately resulting in phosphorylation of the transcription factors IRF-3 and IRF-7 (Ntoufa et al., 2016). After that, IRF-3 and IRF-7 translocate to the nucleus and induce the expression of type 1 interferons and activate the expression of proinflammatory cytokines and chemokines as well (Sato et al., 2003; Landstrom, 2010). In addition, TLR4 utilizes both MYD88-dependent pathway and TRIF pathway (Rana et al., 2016).

TLR7 agonists will become inoperative unless delivered to endosomal vesicles, where the receptor resides. Conjugation of a TLR7 agonist to phospholipids, which is widely applied in drug delivery, can improve its bioavailability as well as the immune response. However, not all of the delivery molecules can induce a comparable response. TLR7 agonists conjugated to mouse serum albumin induced a small quantity of Type 1 interferons. Probably, TLR7 agonists and phospholipid conjugation could facilitate the uptake by pDC and enhance persistence of ligands in endosomal vesicles (Chan et al., 2009). PDC is the major source of Type 1 interferon. It was reported that pDC recognition of pathogens was mainly mediated by TLR7 and TLR9 pathway. Most of the TLR7 agonists have no direct cytotoxic effects on tumor cells. It was reported that deficiency of NK cells might interdict the effects of TLR7 agonists. 3M-011 lost its anti-tumor property in NK1.1-immunodepleted mice, demonstrating the crucial role of NK cells in anti-tumor activities (Dumitru et al., 2009).

There was another discovery which demonstrated that TLR7 activation by imiquimod and resiquimod could induce apoptosis of cancer cells in an assay involving Annexin V-staining. Further study revealed that expression of Bcl-2 was downregulated and cleavage of caspase-3, caspase-7 was upregulated in imiquimod-treated cells (Smits et al., 2010), suggesting that the apoptosis-inducing effects of imiquimod were involved in a caspase-dependent mitochondrial pathway. Futhermore, imiquimod induced reactive oxygen species production to stimulate ATM/ATR pathways and lead to p53-dependent apoptosis in the skin basal cell carcinoma cells (Huang et al., 2016). However, there were no available studies testifying that MYD88 pathway activates p53 expression. The ability of TLR signaling pathway to cross-talk with other pathways is important for inflammatory response (Figure 1; Brown et al., 2011).

Increased regulatory T cells (Tregs) in cancers are adverse to immune therapy. Activation of some TLRs, e.g., TLR4 may stimulate proliferation of Tregs and promote their suppressive function. TLR7 agonist loxoribin was found to modulate CD4+ T cell proliferation and suppress the activity of Tregs via DCs depending on TLR7 pathway (Wang et al., 2014). Additionally, TLRs activation is associated with some autoimmune disorders, such as systemic lupus erythematosus and autoimmune diabetes. Repeated topical treatment of NOD mice with a TLR7 agonist expedited the onset of autoimmune diabetes (Lee et al., 2011). Moreover, functional TLRs are also expressed on some tumors and play an important role in cancer progression. Activation of tumor TLRs may lead to proliferation of tumor cells and enhancement of tumor invasion (Huang et al., 2005, 2008; Cherfils-Vicini et al., 2010).

## Conclusion

TLR 7 agonists are small molecules. They stimulate innate immune cells leading to the activation of humoral and cellular immunity, thus engendering a series of anti-tumor activities. The mechanism of action of TLR7 agonists is associated with the MYD88-dependent pathway and caspase-dependent mitochondrial pathway. Further research on the synthesis of currently available TLR7 agonists may not only shed light on their preclinical pharmacological properties, but also on cancer therapy in the clinical setting.

## Author contributions

HC and CL conceived the topic and drafted the manuscript. FZ edited the manuscript, and LZ drafted the Table. OS, GJ, and TN supervised the work and revised the paper.

### Conflict of interest statement

The authors declare that the research was conducted in the absence of any commercial or financial relationships that could be construed as a potential conflict of interest.

## Abbreviations

NK: natural killer
IDO: indoleamine 2, 3-dioxygenase
HPV: human papillomavirus
pDC: plasmacytoid dendritic cells
Tregs: regulatory T cells.

## References

[B1] Adams S. Kozhaya L. Martiniuk F. Meng T. C. Chiriboga L. Liebes L.. (2012). Topical TLR7 agonist imiquimod can induce immune-mediated rejection of skin metastases in patients with breast cancer. Clin. Cancer Res. 18, 6748–6757. 10.1158/1078-0432.CCR-12-1149 22767669 PMC3580198

[B2] Adlard A. L. Dovedi S. J. Telfer B. A. Koga-Yamakawa E. Pollard C. Honeychurch J.. (2014). A novel systemically administered Toll-like receptor 7 agonist potentiates the effect of ionizing radiation in murine solid tumor models. Int. J. Cancer 135, 820–829. 10.1002/ijc.28711 24390981 PMC4286010

[B3] Agarwala S. S. Kirkwood J. M. Bryant J. (2000). Phase 1, randomized, double-blind trial of 7-allyl-8-oxoguanosine (loxoribine) in advanced cancer. Cytokines Cell. Mol. Ther. 6, 171–176. 10.1080/mccm.6.4.171.176 11565955

[B4] Ahn M. Y. Kwon S. M. Cheong H. H. Park J. H. Lee J. Min S. K.. (2012). Toll-like receptor 7 agonist, imiquimod, inhibits oral squamous carcinoma cells through apoptosis and necrosis. J. Oral Pathol. Med. 41, 540–546. 10.1111/j.1600-0714.2012.01158.x 22577802

[B5] Aurisicchio L. Peruzzi D. Conforti A. Dharmapuri S. Biondo A. Giampaoli S.. (2009). Treatment of mammary carcinomas in HER-2 transgenic mice through combination of genetic vaccine and an agonist of Toll-like receptor 9. Clin. Cancer Res. 15, 1575–1584. 10.1158/1078-0432.CCR-08-2628 19240169

[B6] Ayari C. Besancon M. Bergeron A. LaRue H. Bussieres V. Fradet Y. (2016). Poly(I:C) potentiates Bacillus Calmette-Guerin immunotherapy for bladder cancer. Cancer Immunol. Immunother. 65, 223–234. 10.1007/s00262-015-1789-y 26759009 PMC11029542

[B7] Bam R. A. Hansen D. Irrinki A. Mulato A. Jones G. S. Hesselgesser J.. (2017). TLR7 Agonist GS-9620 Is a Potent Inhibitor of Acute HIV-1 infection in human peripheral blood mononuclear cells. Antimicrob. Agents Chemother. 61. e01369–16. 10.1128/AAC.01369-16 27799218 PMC5192112

[B8] Brody J. D. Ai W. Z. Czerwinski D. K. Torchia J. A. Levy M. Advani R. H.. (2010). *In situ* vaccination with a TLR9 agonist induces systemic lymphoma regression: a phase I/II study. J. Clin. Oncol. 28, 4324–4332. 10.1200/JCO.2010.28.9793 20697067 PMC2954133

[B9] Broomfield S. A. van der Most R. G. Prosser A. C. Mahendran S. Tovey M. G. Smyth M. J.. (2009). Locally administered TLR7 agonists drive systemic antitumor immune responses that are enhanced by anti-CD40 immunotherapy. J. Immunol. 182, 5217–5224. 10.4049/jimmunol.0803826 19380767

[B10] Brown J. Wang H. Hajishengallis G. N. Martin M. (2011). TLR-signaling networks: an integration of adaptor molecules, kinases, and cross-talk. J. Dent. Res. 90, 417–427. 10.1177/0022034510381264 20940366 PMC3075579

[B11] Carvalho J. L. Britto A. de Oliveira A. P. Castro-Faria-Neto H. Albertini R. Anatriello E.. (2016). Beneficial effect of low-level laser therapy in acute lung injury after i-I/R is dependent on the secretion of IL-10 and independent of the TLR/MyD88 signaling. Lasers Med. Sci. 32, 305–315. 10.1007/s10103-016-2115-4 27924419

[B12] Chan M. Hayashi T. Kuy C. S. Gray C. S. Wu C. C. Corr M.. (2009). Synthesis and immunological characterization of toll-like receptor 7 agonistic conjugates. Bioconjug. Chem. 20, 1194–1200. 10.1021/bc900054q 19445505 PMC2976567

[B13] Chen L. Xu Y. Y. Zhou J. M. Wu Y. Y. E Q. Zhu Y. Y. (2012). TLR3 dsRNA agonist inhibits growth and invasion of HepG2.2.15 HCC cells. Oncol. Rep. 28, 200–206. 10.3892/or.2012.1791 22552584

[B14] Cherfils-Vicini J. Platonova S. Gillard M. Laurans L. Validire P. Caliandro R.. (2010). Triggering of TLR7 and TLR8 expressed by human lung cancer cells induces cell survival and chemoresistance. J. Clin. Invest. 120, 1285–1297. 10.1172/JCI36551 20237413 PMC2846035

[B15] Chiba Y. Mizoguchi I. Mitobe K. Higuchi K. Nagai H. Nishigori C.. (2013). IL-27 enhances the expression of TRAIL and TLR3 in human melanomas and inhibits their tumor growth in cooperation with a TLR3 agonist poly(I:C) partly in a TRAIL-dependent manner. PLoS ONE 8:e76159. 10.1371/journal.pone.0076159 24155891 PMC3796519

[B16] Conforti A. Cipriani B. Peruzzi D. Dharmapuri S. Kandimalla E. R. Agrawal S.. (2010). A TLR9 agonist enhances therapeutic effects of telomerase genetic vaccine. Vaccine 28, 3522–3530. 10.1016/j.vaccine.2010.03.020 20332048

[B17] Damiano V. Caputo R. Bianco R. D'Armiento F. P. Leonardi A. De Placido S.. (2006). Novel toll-like receptor 9 agonist induces epidermal growth factor receptor (EGFR) inhibition and synergistic antitumor activity with EGFR inhibitors. Clin. Cancer Res. 12, 577–583. 10.1158/1078-0432.CCR-05-1943 16428503

[B18] Damiano V. Caputo R. Garofalo S. Bianco R. Rosa R. Merola G.. (2007). TLR9 agonist acts by different mechanisms synergizing with bevacizumab in sensitive and cetuximab-resistant colon cancer xenografts. Proc. Natl. Acad. Sci. U.S.A. 104, 12468–12473. 10.1073/pnas.0705226104 17636117 PMC1920540

[B19] Davis M. B. Vasquez-Dunddel D. Fu J. Albesiano E. Pardoll D. Kim Y. J. (2011). Intratumoral administration of TLR4 agonist absorbed into a cellular vector improves antitumor responses. Clin. Cancer Res. 17, 3984–3992. 10.1158/1078-0432.CCR-10-3262 21543518 PMC3117911

[B20] Dewan M. Z. Vanpouille-Box C. Kawashima N. DiNapoli S. Babb J. S. Formenti S. C.. (2012). Synergy of topical toll-like receptor 7 agonist with radiation and low-dose cyclophosphamide in a mouse model of cutaneous breast cancer. Clin. Cancer Res. 18, 6668–6678. 10.1158/1078-0432.CCR-12-0984 23048078 PMC3525760

[B21] Dharmapuri S. Aurisicchio L. Neuner P. Verdirame M. Ciliberto G. La Monica N. (2009). An oral TLR7 agonist is a potent adjuvant of DNA vaccination in transgenic mouse tumor models. Cancer Gene Ther. 16, 462–472. 10.1038/cgt.2008.91 18989354

[B22] Diao Y. Wang X. Wan Y. Zhong J. Gao D. Liu Y.. (2016). Antitumor activity of a novel small molecule TLR7 agonist via immune response induction and tumor microenvironment modulation. Oncol. Rep. 35, 793–800. 10.3892/or.2015.4436 26718332

[B23] Ding L. Ren J. Zhang D. Li Y. Huang X. Ji J.. (2017). The TLR3 Agonist Inhibit Drug efflux and Sequentially Consolidates Low-dose Cisplatin-based Chemoimmunotherapy while Reducing Side effects. Mol. Cancer Ther. 16, 1–12. 10.1158/1535-7163.MCT-16-0454 28138030

[B24] Dovedi S. J. Adlard A. L. Ota Y. Murata M. Sugaru E. Koga-Yamakawa E.. (2016). Intravenous administration of the selective toll-like receptor 7 agonist DSR-29133 leads to anti-tumor efficacy in murine solid tumor models which can be potentiated by combination with fractionated radiotherapy. Oncotarget 7, 17035–17046. 10.18632/oncotarget.7928 26959743 PMC4941369

[B25] Dovedi S. J. Melis M. H. Wilkinson R. W. Adlard A. L. Stratford I. J. Honeychurch J.. (2013). Systemic delivery of a TLR7 agonist in combination with radiation primes durable antitumor immune responses in mouse models of lymphoma. Blood 121, 251–259. 10.1182/blood-2012-05-432393 23086756

[B26] Dumitru C. D. Antonysamy M. A. Gorski K. S. Johnson D. D. Reddy L. G. Lutterman J. L.. (2009). NK1.1+ cells mediate the antitumor effects of a dual Toll-like receptor 7/8 agonist in the disseminated B16-F10 melanoma model. Cancer Immunol. Immunother. 58, 575–587. 10.1007/s00262-008-0581-7 18791716 PMC11030691

[B27] Dummer R. Hauschild A. Becker J. C. Grob J. J. Schadendorf D. Tebbs V.. (2008). An exploratory study of systemic administration of the toll-like receptor-7 agonist 852A in patients with refractory metastatic melanoma. Clin. Cancer Res. 14, 856–864. 10.1158/1078-0432.CCR-07-1938 18245549

[B28] Fahey L. M. Raff A. B. Da Silva D. M. Kast W. M. (2009). Reversal of human papillomavirus-specific T cell immune suppression through TLR agonist treatment of Langerhans cells exposed to human papillomavirus type 16. J. Immunol. 182, 2919–2928. 10.4049/jimmunol.0803645 19234187 PMC2745289

[B29] Fosdick A. Zheng J. Pflanz S. Frey C. R. Hesselgesser J. Halcomb R. L.. (2014). Pharmacokinetic and pharmacodynamic properties of GS-9620, a novel Toll-like receptor 7 agonist, demonstrate interferon-stimulated gene induction without detectable serum interferon at low oral doses. J. Pharmacol. Exp. Ther. 348, 96–105. 10.1124/jpet.113.207878 24133297

[B30] Friedberg J. W. Kelly J. L. Neuberg D. Peterson D. R. Kutok J. L. Salloum R.. (2009). Phase II study of a TLR-9 agonist (1018 ISS) with rituximab in patients with relapsed or refractory follicular lymphoma. Br. J. Haematol. 146, 282–291. 10.1111/j.1365-2141.2009.07773.x 19519691 PMC2747261

[B31] Fujioka T. Ishikura K. Hasegawa M. Ogyu K. Matsushita Y. Sato M.. (1995). Antitumor effects of oral administration of an interferon-inducing pyrimidinone, Bropirimine, on murine renal-cell carcinoma. Cancer Chemother. Pharmacol. 36, 7–12. 10.1007/BF00685725 7536641

[B32] Gableh F. Saeidi M. Hemati S. Hamdi K. Soleimanjahi H. Gorji A.. (2016). Combination of the toll like receptor agonist and alpha-Galactosylceramide as an efficient adjuvant for cancer vaccine. J. Biomed. Sci. 23:16. 10.1186/s12929-016-0238-3 26811064 PMC4727273

[B33] Galli R. Paone A. Fabbri M. Zanesi N. Calore F. Cascione L.. (2013). Toll-like receptor 3 (TLR3) activation induces microRNA-dependent reexpression of functional RARbeta and tumor regression. Proc. Natl. Acad. Sci. U.S.A. 110, 9812–9817. 10.1073/pnas.1304610110 23716670 PMC3683754

[B34] Galluzzi L. Vacchelli E. Eggermont A. Fridman W. H. Galon J. Sautes-Fridman C.. (2012). Trial watch: experimental toll-like receptor agonists for cancer therapy. Oncoimmunology 1, 699–716. 10.4161/onci.20696 22934262 PMC3429574

[B35] Geller M. A. Cooley S. Argenta P. A. Downs L. S. Carson L. F. Judson P. L.. (2010). Toll-like receptor-7 agonist administered subcutaneously in a prolonged dosing schedule in heavily pretreated recurrent breast, ovarian, and cervix cancers. Cancer Immunol. Immunother. 59, 1877–1884. 10.1007/s00262-010-0914-1 20820775 PMC4098785

[B36] Grauer O. M. Molling J. W. Bennink E. Toonen L. W. Sutmuller R. P. Nierkens S.. (2008). TLR ligands in the local treatment of established intracerebral murine gliomas. J. Immunol. 181, 6720–6729. 10.4049/jimmunol.181.10.6720 18981089

[B37] Hamm S. Rath S. Michel S. Baumgartner R. (2009). Cancer immunotherapeutic potential of novel small molecule TLR7 and TLR8 agonists. J. Immunotoxicol. 6, 257–265. 10.3109/15476910903286733 19848448

[B38] Han J. H. Lee J. Jeon S. J. Choi E. S. Cho S. D. Kim B. Y.. (2013). *In vitro* and *in vivo* growth inhibition of prostate cancer by the small molecule imiquimod. Int. J. Oncol. 42, 2087–2093. 10.3892/ijo.2013.1898 23588478

[B39] Harrison L. I. Astry C. Kumar S. Yunis C. (2007). Pharmacokinetics of 852A, an imidazoquinoline Toll-like receptor 7-specific agonist, following intravenous, subcutaneous, and oral administrations in humans. J. Clin. Pharmacol. 47, 962–969. 10.1177/0091270007303766 17660481

[B40] Hayashi T. Crain B. Corr M. Chan M. Cottam H. B. Maj R.. (2010). Intravesical Toll-like receptor 7 agonist R-837: optimization of its formulation in an orthotopic mouse model of bladder cancer. Int. J. Urol. 17, 483–490. 10.1111/j.1442-2042.2010.02503.x 20337728 PMC3876967

[B41] Hennessy E. J. Parker A. E. O'Neill L. A. (2010). Targeting Toll-like receptors: emerging therapeutics? Nat. Rev. Drug Discov. 9, 293–307. 10.1038/nrd3203 20380038

[B42] Higgins M. J. Serrano A. Boateng K. Y. Parsons V. A. Phuong T. Seifert A.. (2016). A Multifaceted role for Myd88-Dependent signaling in progression of murine mammary carcinoma. Breast Cancer (Auckl) 10, 157–167. 10.4137/BCBCR.S40075 27812285 PMC5084708

[B43] Huang B. Zhao J. Li H. He K-L., Chen, Y. Mayer L.. (2005). Toll-like receptors on tumor cells facilitate evasion of immune surveillance. Cancer Res. 65, 5009–5014. 10.1158/0008-5472.CAN-05-0784 15958541

[B44] Huang B. Zhao J. Unkeless J. C. Feng Z. Xiong H. (2008). TLR signaling by tumor and immune cells_ a double-edged sword. Oncogene 27, 218–224. 10.1038/sj.onc.1210904 18176603

[B45] Huang S. J. Hijnen D. Murphy G. F. Kupper T. S. Calarese A. W. Mollet I. G.. (2009). Imiquimod enhances IFN-gamma production and effector function of T cells infiltrating human squamous cell carcinomas of the skin. J. Invest. Dermatol. 129, 2676–2685. 10.1038/jid.2009.151 19516264 PMC2841955

[B46] Huang S. W. Chang S. H. Mu S. W. Jiang H. Y. Wang S. T. Kao J. K.. (2016). Imiquimod activates p53-dependent apoptosis in a human basal cell carcinoma cell line. J. Dermatol. Sci. 81, 182–191. 10.1016/j.jdermsci.2015.12.011 26775629

[B47] Ignatz-Hoover J. J. Wang H. Moreton S. A. Chakrabarti A. Agarwal M. K. Sun K.. (2015). The role of TLR8 signaling in acute myeloid leukemia differentiation. Leukemia 29, 918–926. 10.1038/leu.2014.293 25283842 PMC4387126

[B48] Inglefield J. R. Dumitru C. D. Alkan S. S. Gibson S. J. Lipson K. E. Tomai M. A.. (2008). TLR7 agonist 852A inhibition of tumor cell proliferation is dependent on plasmacytoid dendritic cells and type I IFN. J. Interferon Cytokine Res. 28, 253–263. 10.1089/jir.2007.0097 18439103

[B49] Ito H. Ando T. Arioka Y. Saito K. Seishima M. (2015a). Inhibition of indoleamine 2,3-dioxygenase activity enhances the anti-tumour effects of a Toll-like receptor 7 agonist in an established cancer model. Immunology 144, 621–630. 10.1111/imm.12413 25322876 PMC4368168

[B50] Ito H. Ando T. Ogiso H. Arioka Y. Seishima M. (2015b). Inhibition of induced nitric oxide synthase enhances the anti-tumor effects on cancer immunotherapy using TLR7 agonist in mice. Cancer Immunol. Immunother. 64, 429–436. 10.1007/s00262-014-1644-6 25567751 PMC11029476

[B51] Kim Y. H. Girardi M. Duvic M. Kuzel T. Link B. K. Pinter-Brown L.. (2010). Phase I trial of a Toll-like receptor 9 agonist, PF-3512676 (CPG 7909), in patients with treatment-refractory, cutaneous T-cell lymphoma. J. Am. Acad. Dermatol. 63, 975–983. 10.1016/j.jaad.2009.12.052 20888065

[B52] Koga-Yamakawa E. Dovedi S. J. Murata M. Matsui H. Leishman A. J. Bell J.. (2013). Intratracheal and oral administration of SM-276001: a selective TLR7 agonist, leads to antitumor efficacy in primary and metastatic models of cancer. Int. J. Cancer 132, 580–590. 10.1002/ijc.27691 22733292

[B53] Koga-Yamakawa E. Murata M. Dovedi S. J. Wilkinson R. W. Ota Y. Umehara H.. (2015). TLR7 tolerance is independent of the type I IFN pathway and leads to loss of anti-tumor efficacy in mice. Cancer Immunol. Immunother. 64, 1229–1239. 10.1007/s00262-015-1730-4 26091797 PMC11029383

[B54] Landstrom M. (2010). The TAK1-TRAF6 signalling pathway. Int. J. Biochem. Cell Biol. 42, 585–589. 10.1016/j.biocel.2009.12.023 20060931

[B55] Lanford R. E. Guerra B. Chavez D. Giavedoni L. Hodara V. L. Brasky K. M.. (2013). GS-9620, an oral agonist of Toll-like receptor-7, induces prolonged suppression of hepatitis B virus in chronically infected chimpanzees. Gastroenterology 144, 1508–1517, 1517.e1501–e1510. 10.1053/j.gastro.2013.02.003 23415804 PMC3691056

[B56] Lee S. M. Ghoreishi W. K. Cheng T.-Y. E. Chang Y. Q. Zhang J. P. D. (2011). Toll-like receptor 7 stimulation promotes autoimmune. Diabetologia. 54, 1407–1416. 10.1007/s00125-011-2083-y 21340621

[B57] Leigh N. D. Bian G. Ding X. Liu H. Aygun-Sunar S. Burdelya L. G.. (2014). A flagellin-derived toll-like receptor 5 agonist stimulates cytotoxic lymphocyte-mediated tumor immunity. PLoS ONE 9:e85587. 10.1371/journal.pone.0085587 24454895 PMC3891810

[B58] Le Noci V. Tortoreto M. Gulino A. Storti C. Bianchi F. Zaffaroni N.. (2015). Poly(I:C) and CpG-ODN combined aerosolization to treat lung metastases and counter the immunosuppressive microenvironment. Oncoimmunology 4:e1040214. 10.1080/2162402X.2015.1040214 26451303 PMC4589046

[B59] Love E. M. Manalo I. F. Chen S. C. Chen K. H. Stoff B. K. (2016). A video-based educational pilot for basal cell carcinoma (BCC) treatment: A randomized controlled trial. J. Am. Acad. Dermatol. 74, 477–483.e477. 10.1016/j.jaad.2015.10.014 26777101

[B60] Lu H. Wagner W. M. Gad E. Yang Y. Duan H. Amon L. M.. (2010). Treatment failure of a TLR-7 agonist occurs due to self-regulation of acute inflammation and can be overcome by IL-10 blockade. J. Immunol. 184, 5360–5367. 10.4049/jimmunol.0902997 20308630

[B61] Ma F. Zhang J. Zhang J. Zhang C. (2010). The TLR7 agonists imiquimod and gardiquimod improve DC-based immunotherapy for melanoma in mice. Cell. Mol. Immunol. 7, 381–388. 10.1038/cmi.2010.30 20543857 PMC4002679

[B62] Maeda K. Akira S. (2016). TLR7 Structure: Cut in Z-Loop. Immunity 45, 705–707. 10.1016/j.immuni.2016.10.003 27760331

[B63] Martino M. M. Maruyama K. Kuhn G. A. Satoh T. Takeuchi O. Muller R.. (2016). Inhibition of IL-1R1/MyD88 signalling promotes mesenchymal stem cell-driven tissue regeneration. Nat. Commun. 7:11051. 10.1038/ncomms11051 27001940 PMC4804175

[B64] Mauldin I. S. Wages N. A. Stowman A. M. Wang E. Olson W. C. Deacon D. H.. (2016). Topical treatment of melanoma metastases with imiquimod, plus administration of a cancer vaccine, promotes immune signatures in the metastases. Cancer Immunol. Immunother. 65, 1201–1212. 10.1007/s00262-016-1880-z 27522582 PMC5037037

[B65] Nakamura T. Wada H. Kurebayashi H. McInally T. Bonnert R. Isobe Y. (2013). Synthesis and evaluation of 8-oxoadenine derivatives as potent Toll-like receptor 7 agonists with high water solubility. Bioorg. Med. Chem. Lett. 23, 669–672. 10.1016/j.bmcl.2012.11.114 23265901

[B66] Narayan R. Nguyen H. Bentow J. J. Moy L. Lee D. K. Greger S.. (2012). Immunomodulation by imiquimod in patients with high-risk primary melanoma. J. Invest. Dermatol. 132, 163–169. 10.1038/jid.2011.247 21850019 PMC3229834

[B67] Northfelt D. W. Ramanathan R. K. Cohen P. A. Von Hoff D. D. Weiss G. J. Dietsch G. N.. (2014). A phase I dose-finding study of the novel Toll-like receptor 8 agonist VTX-2337 in adult subjects with advanced solid tumors or lymphoma. Clin. Cancer Res. 20, 3683–3691. 10.1158/1078-0432.CCR-14-0392 24807889

[B68] Ntoufa S. Vilia M. G. Stamatopoulos K. Ghia P. Muzio M. (2016). Toll-like receptors signaling: a complex network for NF-kappaB activation in B-cell lymphoid malignancies. Semin. Cancer Biol. 39, 15–25. 10.1016/j.semcancer.2016.07.001 27402288

[B69] Oldford S. A. Haidl I. D. Howatt M. A. Leiva C. A. Johnston B. Marshall J. S. (2010). A critical role for mast cells and mast cell-derived IL-6 in TLR2-mediated inhibition of tumor growth. J. Immunol. 185, 7067–7076. 10.4049/jimmunol.1001137 21041732

[B70] O'Neill L. A. Golenbock D. Bowie A. G. (2013). The history of Toll-like receptors–redefining innate immunity. Nat. Rev. Immunol. 13, 453–460. 10.1038/nri3446 23681101

[B71] Paone A. Galli R. Gabellini C. Lukashev D. Starace D. Gorlach A.. (2010). Toll-like receptor 3 regulates angiogenesis and apoptosis in prostate cancer cell lines through hypoxia-inducible factor 1 alpha. Neoplasia 12, 539–549. 10.1593/neo.92106 20651983 PMC2907580

[B72] Park S. N. Noh K. T. Jeong Y. I. Jung I. D. Kang H. K. Cha G. S.. (2013). Rhamnogalacturonan II is a Toll-like receptor 4 agonist that inhibits tumor growth by activating dendritic cell-mediated CD8+ T cells. Exp. Mol. Med. 45:e8. 10.1038/emm.2013.14 23392255 PMC3584663

[B73] Pockros P. J. Guyader D. Patton H. Tong J. M. Wright T. McHutchison J. G.. (2007). Oral resiquimod in chronic HCV infection_ Safety and efficacy in.pdf. J. Hepatol. 47, 174–182. 10.1016/j.jhep.2007.02.025 17532523

[B74] Pellacani A. Tosi P. Zinzani P. L. Ottaviani E. Albertini P. Magagnoli M.. (1999). Cytotoxic combination of loxoribine with fludarabine and mafosfamide on freshly isolated B-chronic lymphocytic leukemia cells. Leuk. Lymphoma 33, 147–153. 10.3109/10428199909093736 10194132

[B75] Pham T. N. Hong C. Y. Min J. J. Rhee J. H. Nguyen T. A. Park B. C.. (2010). Enhancement of antitumor effect using dendritic cells activated with natural killer cells in the presence of Toll-like receptor agonist. Exp. Mol. Med. 42, 407–419. 10.3858/emm.2010.42.6.042 20386085 PMC2892594

[B76] Piccinini A. M. Midwood K. S. (2010). DAMPening inflammation by modulating TLR signalling. Mediat. Inflamm. 2010:672395. 10.1155/2010/672395 20706656 PMC2913853

[B77] Pope B. L. Sigindere J. Chourmouzis E. MacIntyre P. Goodman M. G. (1994). 7-Allyl-8-oxoguanosine (loxoribine) inhibits the metastasis of B16 melanoma cells and has adjuvant activity in mice immunized with a B16 tumor vaccine. Cancer Immunol. Immunother. 38, 83–91. 10.1007/BF01526202 8306370 PMC11038224

[B78] Pradhan P. Qin H. Leleux J. A. Gwak D. Sakamaki I. Kwak L. W.. (2014). The effect of combined IL10 siRNA and CpG ODN as pathogen-mimicking microparticles on Th1/Th2 cytokine balance in dendritic cells and protective immunity against B cell lymphoma. Biomaterials 35, 5491–5504. 10.1016/j.biomaterials.2014.03.039 24720881 PMC4747034

[B79] Qu J. Hou Z. Han Q. Zhang C. Tian Z. Zhang J. (2013). Poly(I:C) exhibits an anti-cancer effect in human gastric adenocarcinoma cells which is dependent on RLRs. Int. Immunopharmacol. 17, 814–820. 10.1016/j.intimp.2013.08.013 24029594

[B80] Rana M. Maurya P. Reddy S. S. Singh V. Ahmad H. Dwivedi A. K.. (2016). A Standardized Chemically Modified Curcuma longa Extract Modulates IRAK-MAPK Signaling in Inflammation and Potentiates Cytotoxicity. Front. Pharmacol. 7:223. 10.3389/fphar.2016.00223 27504095 PMC4959270

[B81] Ren T. Xu L. Jiao S. Wang Y. Cai Y. Liang Y.. (2009). TLR9 signaling promotes tumor progression of human lung cancer cell *in vivo*. Pathol. Oncol. Res. 15, 623–630. 10.1007/s12253-009-9162-0 19319670

[B82] Rook A. H. Gelfand J. M. Wysocka M. Troxel A. B. Benoit B. Surber C.. (2015). Topical resiquimod can induce disease regression and enhance T-cell effector functions in cutaneous T-cell lymphoma. Blood 126, 1452–1461. 10.1182/blood-2015-02-630335 26228486 PMC4573868

[B83] Rosa R. Melisi D. Damiano V. Bianco R. Garofalo S. Gelardi T.. (2011). Toll-like receptor 9 agonist IMO cooperates with cetuximab in K-ras mutant colorectal and pancreatic cancers. Clin. Cancer Res. 17, 6531–6541. 10.1158/1078-0432.CCR-10-3376 21890455

[B84] Sabado R. L. Pavlick A. Gnjatic S. Cruz C. M. Vengco I. Hasan F.. (2015). Resiquimod as an immunologic adjuvant for NY-ESO-1 protein vaccination in patients with high-risk melanoma. Cancer Immunol Res 3, 278–287. 10.1158/2326-6066.CIR-14-0202 25633712 PMC4374362

[B85] Salazar L. G. Lu H. Reichow J. L. Childs J. S. Coveler A. L. Higgins D. M.. (2017). Topical imiquimod plus nab-paclitaxel for breast cancer cutaneous metastases: a phase 2 clinical trial. JAMA Oncol. 10.1001/jamaoncol.2016.6007. [Epub ahead of print]. 28114604 PMC5824239

[B86] Sarosdy M. F. (1997). Oral bropirimine immunotherapy of rodent prostate cancer. Eur. Urol. 31(Suppl. 1), 5–9. 9076480 10.1159/000474525

[B87] Sarosdy M. F. Tangen C. M. Weiss G. R. Nestok B. R. Benson M. C. Schellhammer P. F.. (2005). A phase II clinical trial of oral bropirimine in combination with intravesical bacillus Calmette-Guerin for carcinoma *in situ* of the bladder: a Southwest Oncology Group Study. Urol. Oncol. 23, 386–389. 10.1016/j.urolonc.2005.05.028 16301114 PMC3632328

[B88] Sato S. Sugiyama M. Yamamoto M. Watanabe Y. Kawai T. Takeda K.. (2003). Toll/IL-1 receptor domain-containing adaptor inducing IFN-beta (TRIF) associates with TNF receptor-associated factor 6 and TANK-binding kinase 1, and activates two distinct transcription factors, NF-kappa B and IFN-regulatory factor-3, in the Toll-like receptor signaling. J. Immunol. 171, 4304–4310. 10.4049/jimmunol.171.8.4304 14530355

[B89] Scholch S. Rauber C. Tietz A. Rahbari N. N. Bork U. Schmidt T.. (2015). Radiotherapy combined with TLR7/8 activation induces strong immune responses against gastrointestinal tumors. Oncotarget 6, 4663–4676. 10.18632/oncotarget.3081 25609199 PMC4467106

[B90] Shcheblyakov D. V. Logunov D. Y. Rakovskaya I. V. Shmarov M. M. Naroditsky B. S. Ginzburg A. L. (2011). Triggering of Toll-like Receptor-2 in Mouse Myelomonocytic Leukemia Cells WEHI-3B Leads to the suppression of apoptosis and promotes tumor progression *in vivo*. Acta Nat. 3, 83–93. 22649707 PMC3347616

[B91] Singh M. Khong H. Dai Z. Huang X. F. Wargo J. A. Cooper Z. A.. (2014). Effective innate and adaptive antimelanoma immunity through localized TLR7/8 activation. J. Immunol. 193, 4722–4731. 10.4049/jimmunol.1401160 25252955 PMC4201984

[B92] Smith D. A. Conkling P. Richards D. A. Nemunaitis J. J. Boyd T. E. Mita A. C.. (2014). Antitumor activity and safety of combination therapy with the Toll-like receptor 9 agonist IMO-2055, erlotinib, and bevacizumab in advanced or metastatic non-small cell lung cancer patients who have progressed following chemotherapy. Cancer Immunol. Immunother. 63, 787–796. 10.1007/s00262-014-1547-6 24770667 PMC11028443

[B93] Smits E. L. Cools N. Lion E. Van Camp K. Ponsaerts P. Berneman Z. N.. (2010). The Toll-like receptor 7/8 agonist resiquimod greatly increases the immunostimulatory capacity of human acute myeloid leukemia cells. Cancer Immunol. Immunother. 59, 35–46. 10.1007/s00262-009-0721-8 19449004 PMC11029891

[B94] Spaner D. E. Shi Y. White D. Shaha S. He L. Masellis A.. (2010). A phase I/II trial of TLR-7 agonist immunotherapy in chronic lymphocytic leukemia. Leukemia 24, 222–226. 10.1038/leu.2009.195 19759558

[B95] Stone G. W. Barzee S. Snarsky V. Santucci C. Tran B. Kornbluth R. S. (2009a). Regression of established AB1 murine mesothelioma induced by peritumoral injections of CpG oligodeoxynucleotide either alone or in combination with poly(I:C) and CD40 ligand plasmid DNA. J. Thorac. Oncol. 4, 802–808. 10.1097/JTO.0b013e3181a8634d 19550243

[B96] Stone G. W. Barzee S. Snarsky V. Santucci C. Tran B. Langer R.. (2009b). Nanoparticle-delivered multimeric soluble CD40L DNA combined with Toll-Like Receptor agonists as a treatment for melanoma. PLoS ONE 4:e7334. 10.1371/journal.pone.0007334 19812695 PMC2754331

[B97] Tei Y. Matsuyama H. Wada T. Kurisu H. Tahara M. Naito K. (2002). *In vitro* antitumor activity of bropirimine against urinary bladder tumor cells. Anticancer Res. 22, 1667–1671. 12168852

[B98] Tosi P. Zinzani P. L. Pellacani A. Ottaviani E. Magagnoli M. Tura S. (1997). Loxoribine affects fludarabine activity on freshly isolated B-chronic lymphocytic leukemia cells. Leuk. Lymphoma 26, 343–348. 10.3109/10428199709051784 9322897

[B99] Ursu R. Taillibert S. Banissi C. Vicaut E. Bailon O. Le Rhun E.. (2015). Immunotherapy with CpG-ODN in neoplastic meningitis: a phase I trial. Cancer Sci. 106, 1212–1218. 10.1111/cas.12724 26094710 PMC4582991

[B100] Vacchelli E. Eggermont A. Sautes-Fridman C. Galon J. Zitvogel L. Kroemer G.. (2013). Trial Watch: Toll-like receptor agonists for cancer therapy. Oncoimmunology 2:e25238. 10.4161/onci.25238 24083080 PMC3782517

[B101] Vacchelli E. Galluzzi L. Eggermont A. Fridman W. H. Galon J. Sautes-Fridman C.. (2012). Trial watch: FDA-approved Toll-like receptor agonists for cancer therapy. Oncoimmunology 1, 894–907. 10.4161/onci.20931 23162757 PMC3489745

[B102] Wang S. Astsaturov I. A. Bingham C. A. McCarthy K. M. von Mehren M. Xu W.. (2012). Effective antibody therapy induces host-protective antitumor immunity that is augmented by TLR4 agonist treatment. Cancer Immunol. Immunother. 61, 49–61. 10.1007/s00262-011-1090-7 21842208 PMC3517883

[B103] Wang C. Zhou Q. Wang X. Wu X. Chen X. Jianfang Li Z. Z. (2014). The TLR7 agonist induces tumor regression both by promoting CD4^+^T cells proliferation and by reversing T regulatory cell-mediated suppression via dendritic cells. Oncotarget 6, 1779–1789. 10.18632/oncotarget.2757 PMC4359331 25593198

[B104] Wang X. D. Gao N. N. Diao Y. W. Liu Y. Gao D. Li W.. (2015). Conjugation of toll-like receptor-7 agonist to gastric cancer antigen MG7-Ag exerts antitumor effects. World J. Gastroenterol. 21, 8052–8060. 10.3748/wjg.v21.i26.8052 26185376 PMC4499347

[B105] Weber A. Zimmermann C. Mausberg A. K. Kieseier B. C. Hartung H. P. Hofstetter H. H. (2013). Induction of pro-inflammatory cytokine production in thymocytes by the immune response modifiers Imiquimod and Gardiquimod. Int. Immunopharmacol. 17, 427–431. 10.1016/j.intimp.2013.06.023 23867290

[B106] Wei J. Zang S. Xu M. Zheng Q. Chen X. Qin Q. (2017). TRAF6 is a critical factor in fish immune response to virus infection. Fish Shellfish Immunol. 60, 6–12. 10.1016/j.fsi.2016.11.008 27818344

[B107] Weigel B. J. Cooley S. DeFor T. Weisdorf D. J. Panoskaltsis-Mortari A. Chen W.. (2012). Prolonged subcutaneous administration of 852A, a novel systemic toll-like receptor 7 agonist, to activate innate immune responses in patients with advanced hematologic malignancies. Am. J. Hematol. 87, 953–956. 10.1002/ajh.23280 22718533 PMC3638918

[B108] Wiedemann G. M. Jacobi S. J. Chaloupka M. Krachan A. Hamm S. Strobl S.. (2016). A novel TLR7 agonist reverses NK cell anergy and cures RMA-S lymphoma-bearing mice. Oncoimmunology 5:e1189051. 10.1080/2162402X.2016.1189051 27622045 PMC5006928

[B109] Wu J. Feldman R. Barry G. T. Kulp J. Adams M. P. Levy S. (2012). Pharmacokinetics of Daily Self-Application. J. Clin. Pharmacol. 52, 828–836. 10.1177/0091270011407192 22232733

[B110] Xu Y. Y. Chen L. Zhou J. M. Wu Y. Y. Zhu Y. Y. (2013). Inhibitory effect of dsRNA TLR3 agonist in a rat hepatocellular carcinoma model. Mol. Med. Rep. 8, 1037–1042. 10.3892/mmr.2013.1646. 23970360

[B111] Yin T. He S. Wang Y. (2015). Toll-like receptor 7/8 agonist, R848, exhibits antitumoral effects in a breast cancer model. Mol. Med. Rep. 12, 3515–3520. 10.3892/mmr.2015.3885 26043701

[B112] Zhang Y. Lin A. Zhang C. Tian Z. Zhang J. (2014). Phosphorothioate-modified CpG oligodeoxynucleotide (CpG ODN) induces apoptosis of human hepatocellular carcinoma cells independent of TLR9. Cancer Immunol. Immunother. 63, 357–367. 10.1007/s00262-014-1518-y 24452201 PMC11029435

[B113] Zhang Y. Luo F. Cai Y. Liu N. Wang L. Xu D.. (2011). TLR1/TLR2 agonist induces tumor regression by reciprocal modulation of effector and regulatory T cells. J. Immunol. 186, 1963–1969. 10.4049/jimmunol.1002320 21217015

[B114] Zhou S. X. Li F. S. Qiao Y. L. Zhang X. Q. Wang Z. D. (2012). Toll-like receptor 5 agonist inhibition of growth of A549 lung cancer cells *in vivo* in a Myd88 dependent manner. Asian Pac. J. Cancer Prev. 13, 2807–2812. 10.7314/APJCP.2012.13.6.2807 22938463

[B115] Zhu J. He S. Du J. Wang Z. Li W. Chen X.. (2015). Local administration of a novel Toll-like receptor 7 agonist in combination with doxorubicin induces durable tumouricidal effects in a murine model of T cell lymphoma. J. Hematol. Oncol. 8:21. 10.1186/s13045-015-0121-9 25887995 PMC4359787

[B116] Zou B. B. Wang F. Li L. Cheng F. W. Jin R. Luo X.. (2015). Activation of Toll-like receptor 7 inhibits the proliferation and migration, and induces the apoptosis of pancreatic cancer cells. Mol. Med. Rep. 12, 6079–6085. 10.3892/mmr.2015.4130 26238718

[B117] Zou P. F. Huang X. N. Yao C. L. Sun Q. X. Li Y. Zhu Q.. (2016). Cloning and functional characterization of IRAK4 in large yellow croaker (Larimichthys crocea) that associates with MyD88 but impairs NF-kappaB activation. Fish Shellfish Immunol. 63, 452–464. 10.1016/j.fsi.2016.12.019 27989863

